# Impact of elevated CO_2_ level and egg quiescence duration on gene expression in the peripheral olfactory system of *Aedes aegypti*

**DOI:** 10.1038/s41598-025-98159-w

**Published:** 2025-04-24

**Authors:** Sukritha Nalikkaramal, Sharon Rose Hill, Rickard Ignell

**Affiliations:** 1 Department of Plant Protection Biology, Disease Vector Group, Box 102 234 56, Lomma, Alnarp, Sweden; 2 Max Planck Center Next Generation Insect Chemical Ecology, Alnarp, Sweden

**Keywords:** Mosquitoes, Carbon dioxide, Climate change, Egg quiescence, Olfactory system, Transcriptome, Behavioural ecology, Climate-change ecology, Molecular ecology

## Abstract

Elevation in CO_2_ can significantly impact the biology of various organisms, affecting life-history traits of both aquatic and terrestrial forms, including disease-vectoring mosquitoes. For mosquitoes, this effect is accentuated by egg quiescence duration, resulting in a change in foraging of adult females. Female mosquitoes rely on their olfactory system for locating resources, such as nectar and blood. This study employs a transcriptomic approach to investigate how a projected elevation in CO_2_ level, under a worst-case scenario, interacts with extended egg quiescence duration to modulate the molecular machinery of the peripheral olfactory system, the antennae and maxillary palps, of the yellow fever mosquito, *Aedes aegypti*. The transcriptome analysis demonstrates significant changes in the abundance of genes related to metabolism, xenobiotics degradation and chemosensory function, with the most pronounced effects observed in the CO_2_ sensing tissue, the maxillary palp. The study provides novel insights into how anthropogenic climate change can modulate the olfactory sensory system of disease vectors, which may have cascading effects on resource-seeking behaviour.

## Introduction

Global climate change, due to anthropogenic activities, is predicted to change the distribution and behaviour of insects, including mosquitoes that vector disease^1–3^. A key factor driving this change is the elevation in atmospheric carbon dioxide (CO_2_), which inadvertently affects life history traits across both aquatic and terrestrial stages of invertebrates^4–6^. For example, in the dengue vector, *Aedes aegypti,* an exponential increase in atmospheric CO_2_ level, reflecting those projected within recent time and those predicted under extreme conditions, if targets are not met, for the next century^7^, significantly affects key life-history traits, including larval survival and development, as well as adult survival and the feeding response of females^6^. These effects are further modulated by the extent of egg quiescence, *i.e.,* the ability of eggs to withstand extended periods of desiccation or dormancy^8^, which is determined by environmental factors, such as temperature and humidity^9–11^. Prolonged egg quiescent duration increases the susceptibility of emerging larvae to abiotic stressors^12,13^, which may have significant effects on mosquito population dynamics and feeding behaviour, thus affecting vectorial capacity^14^. The feeding response of insects is the ultimate stage in a process regulating resource seeking, which for most insects is mediated predominantly by olfaction and influenced by the internal physiological state^15,16^. Resource-seeking behaviours, as well as the detection of ecologically relevant sensory cues, in insects are affected by both short- and long-term exposure to elevated levels of CO_2_^17–21^.

Many insect species use CO_2_ as a reliable cue for nectar^22,23^, host^24,25^, and oviposition site seeking^26^, as well as threat avoidance^27,28^. An elevation in ambient CO_2_ negatively affects host-seeking in mosquitoes^21^ and oviposition site selection in moths, due to sensory constraints imposed on the CO_2_-sensory system^21,26^. Long-term developmental exposure to elevated CO_2_ also reduces the alarm-pheromone escape behaviour in aphids^29^, although no significant effects on olfactory perception have been described^30^. In addition, studies on aquatic invertebrates show an impairment in olfactory-guided behaviours as a consequence of elevated CO_2_^31^, however, the underlying neuronal mechanism remains unclear^32^. Exposure to elevated CO_2_ in *Helicoverpa* moths affects the CO_2_-sensory neurons, which become promiscuous and respond to fluctuations in temperature, as well as to CO_2_^33^, demonstrating that exposure to elevated CO_2_ likely has a broad effect on sensory systems and gene expression^34^. The aim of this study was to assess how predicted levels of elevated CO_2_ and extended egg quiescence affect chemosensory gene expression to identify molecular correlates underlying changes in resource-seeking behaviours in female *Ae. aegypti*.

The antennae and maxillary palps constitute the main peripheral olfactory system of mosquitoes, with hair-like structures, sensilla, on the surface acting as the smallest functional units^35^. Volatile odorants enter the sensilla, where they are recognized and transported by odorant binding proteins (OBPs) and chemosensory proteins (CSPs) to receptors in the dendritic membrane of olfactory sensory neurons (OSNs)^36,37^. Each OSN expresses one or a combination of olfactory receptor proteins from three different families: odorant receptors (ORs), ionotropic receptors (IRs) and gustatory receptors (GRs)^38,39^, as well as sensory neuron membrane proteins (SNMPs)^36^. The ORs and IRs form heterotetrameric complexes with conserved co-receptors, Orco, as well as Ir25a, Ir8a and Ir76b, respectively, and ligand-selective subunits, ORs and IRs^40,41^. The overall role of ORs and IRs in mosquitoes is to regulate host attraction and discrimination^42–45^. Although GRs are primarily involved in contact chemoreception, CO_2_ is detected by a heteromeric complex of Grs^46,47^, and involved in activation and attraction^48,49^. Apart from the canonical chemosensory gene families, pickpocket (PPK) and transient receptor potential (TRP) channels, involved in risk assessment^50–52^, are also expressed in the OSN dendritic membrane^37^. Several members of these chemosensory gene families are differentially regulated in response to a change in internal state of female mosquitoes^16,53–55^, however, there is currently limited information on how the external environment modulates the molecular machinery of the peripheral olfactory system.

To achieve the aim of this study, RNA sequencing was performed using antennal and maxillary palp tissues collected from females reared under current ambient and extreme CO_2_ conditions and originating from eggs following different egg quiescent periods. The transcriptome analysis demonstrated an overall effect on the differential expression within select gene ontologies, including metabolism, xenobiotics and chemosensory, predominantly in the maxillary palp, in response to elevated CO_2_ conditions, an affect exacerbated by egg quiescence duration. The findings of this study demonstrate that predicted changes in climate, driven by factors, such as elevation in CO_2_, affect the peripheral olfactory system of insects, which in turn may affect the resource-seeking behaviours.

## Results

### RNA sequencing

The RNA sequencing detected a total of 17,439 genes of the 19,804 annotated genes in the genome of *Ae. aegypti*, of which 10,226 were reliably expressed (Supplementary Table S1). Of these, 8,833 and 9,510 were reliably expressed in the antennae and maxillary palps, respectively. To assess the quality and depth of the sequencing, the core eukaryotic gene mapping approach was performed, demonstrating that 450 and 447 (of the total 450) genes were detected reliably above the 1 TPM expression level in the antennal and maxillary palp libraries, respectively (Supplementary Table S2).

### Overall and differential expression

**Fig. 1:**
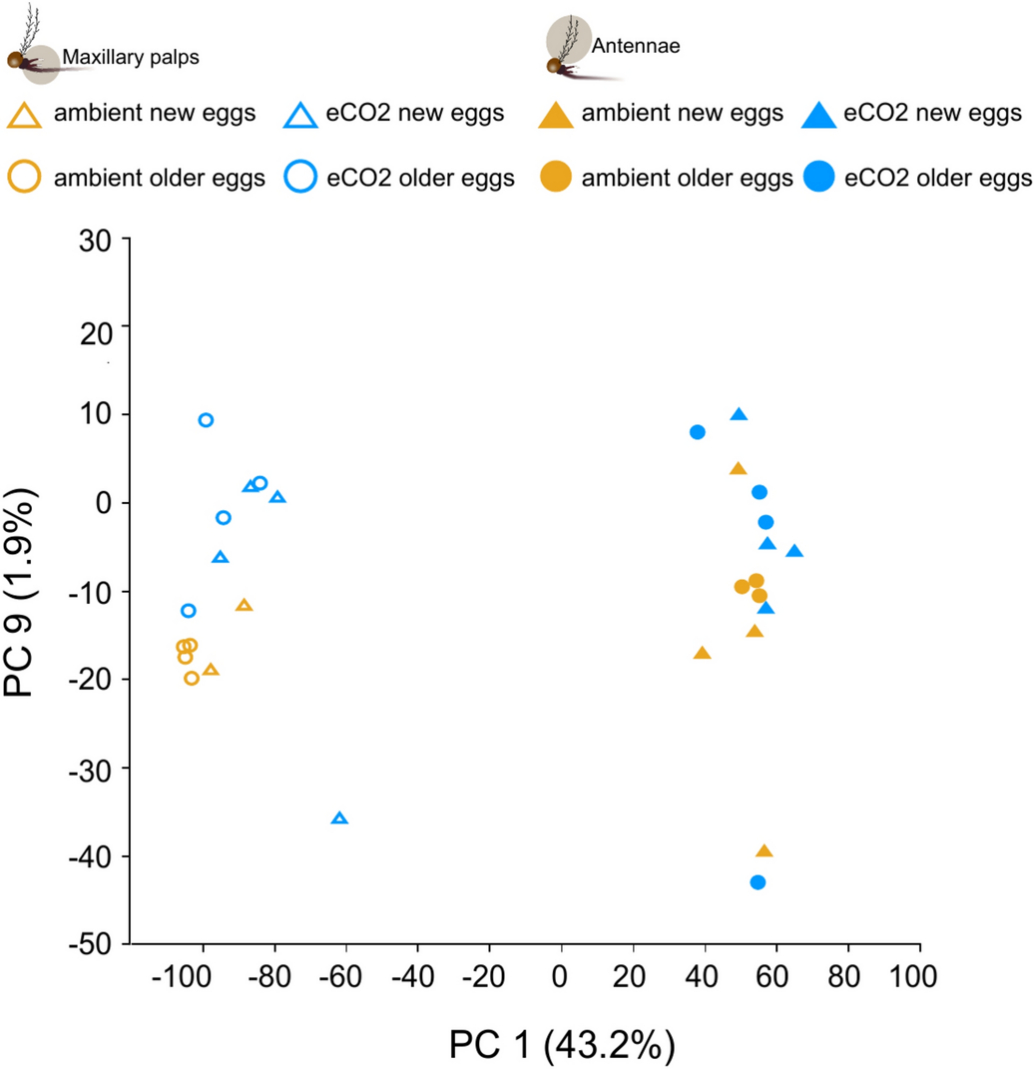
Elevated CO_2_, but not egg quiescence, differentially affects overall gene expression in the peripheral olfactory organs of *Aedes aegypti*. Principal component analysis of antennal and maxillary palp libraries of females emerging from new and older eggs, with short and extended egg quiescent duration, respectively, reared under ambient and elevated CO_2_ conditions. A total of 29 libraries were analysed to estimate the change in overall gene expression, in which Principal Component (PC) 1 (43.2%) and PC 9 (1.9%) accounted for the variance between the libraries.

Overall gene expression was assessed using Principal Component Analysis (PCA) with the 29 libraries of tissue collected from females reared under ambient and elevated CO_2_ conditions, and shorter and extended egg quiescent periods (Fig. 1). The analysis revealed that 43.2% of the variance among libraries was based on the type of olfactory organ (PC 1), and 1.9% of the variance between maxillary palp libraries was based on CO_2_ condition (PC 9) (Fig. 1). There was no significant effect on overall antennal gene expression in response to CO_2_ level (F = 1.01, R^2^ = 0.064, p = 0.38) or egg quiescent duration (F = 1.68, R^2^ = 0.10, p = 0.17), individually or interactively (F = 2.07, R^2^ = 0.13, p = 0.09, Supplementary Figure S1). In contrast, the egg quiescence period (F = 3.43, R^2^ = 0.20, p = 0.02) significantly affected the overall gene expression in the maxillary palp (Supplementary Figure S1). However, neither CO_2_ level (F = 1.01, R^2^ = 0.06, p = 0.38) nor the interaction of the two stress factors (F = 2.07, R^2^ = 0.13, p = 0.09) had a significant effect on the overall maxillary palp gene expression (Supplementary Figure S1).

**Fig. 2:**
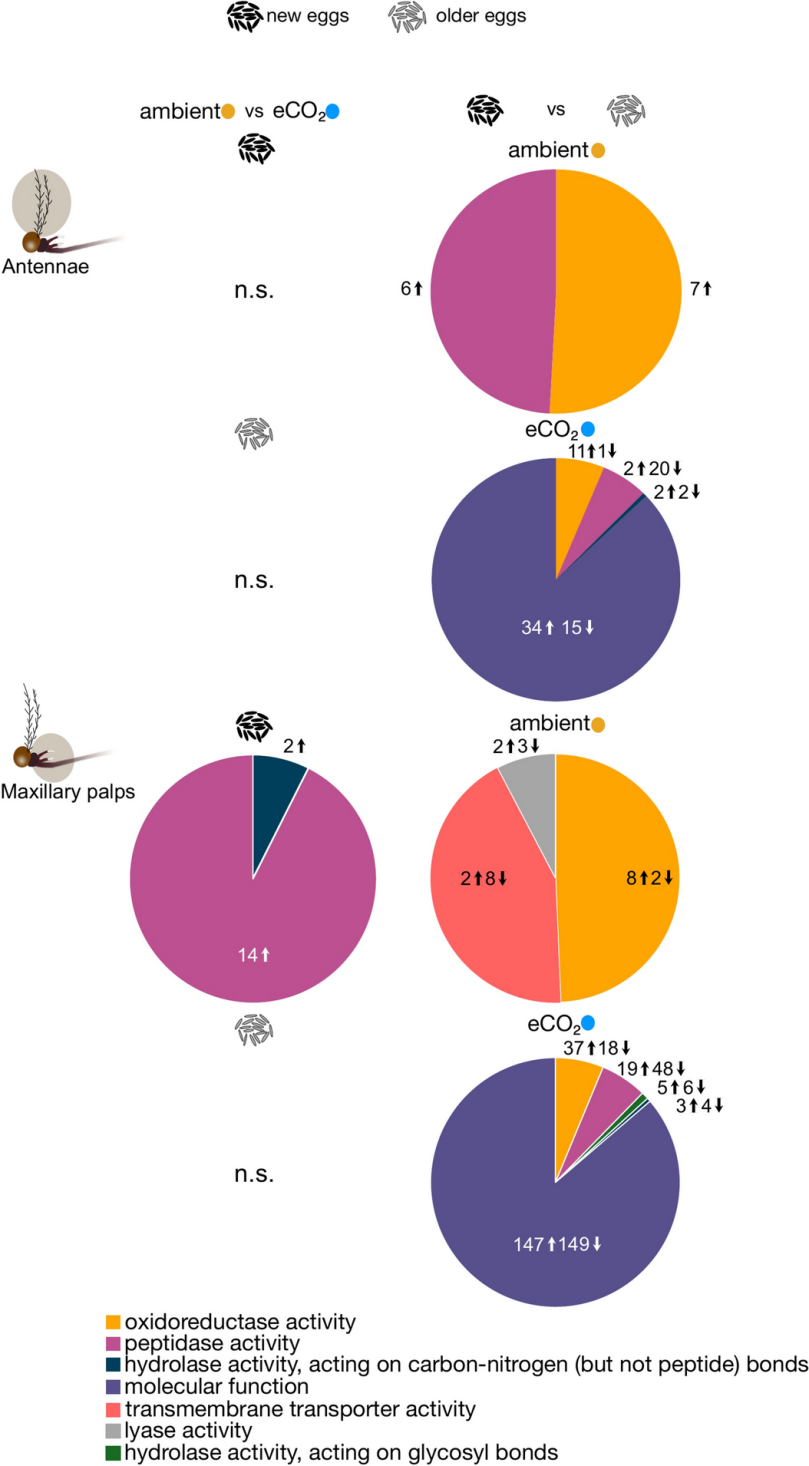
Gene ontology analysis of differentially expressed genes in the antennae and maxillary palps of *Aedes aegypti.* The olfactory tissues were collected from females reared under ambient and elevated CO_2_ conditions, as well as short and extended egg quiescence duration, referred to as new and older eggs, respectively. Pairwise comparisons are arranged in a matrix in response to CO_2_ conditions and egg quiescence period. The differentially expressed genes are classified into molecular function ontology, using gene ontology slim categorisation. n.s.: non-significant.

The gene ontology (GO) analysis identified various molecular functional categories, based on differentially expressed genes (DEGs), which changed in both numbers and direction in the antennal and maxillary palp libraries in response to the interaction of an elevation in CO_2_ and extended egg quiescence (Fig. 2). Comparisons between ambient and elevated CO_2,_ as well as between egg quiescence periods for antennal and maxillary palp libraries under ambient conditions, identified too few DEGs for drawing any overall findings (Fig. 2). In response to elevated CO_2_ and extended egg quiescence, > 85% of the DEGs in the antennal and maxillary palp libraries were categorised as molecular function (GO:0,003,674), followed by oxidoreductase activity (GO:0,016,491), peptidase activity (GO:0,008,233) and hydrolase activity, acting on carbon–nitrogen (but not peptide) bonds (GO:0,016,810) (Fig. 2, right). In addition, in the maxillary palp libraries, the 1% DEGs were categorised as hydrolase activity, acting on glycosyl bonds (GO:0,016,798) (Fig. 2). Within the molecular function category, several differentially expressed chemosensory genes, including *Or*s, *Ir*s and *Obp*s, were represented.

**Fig. 3:**
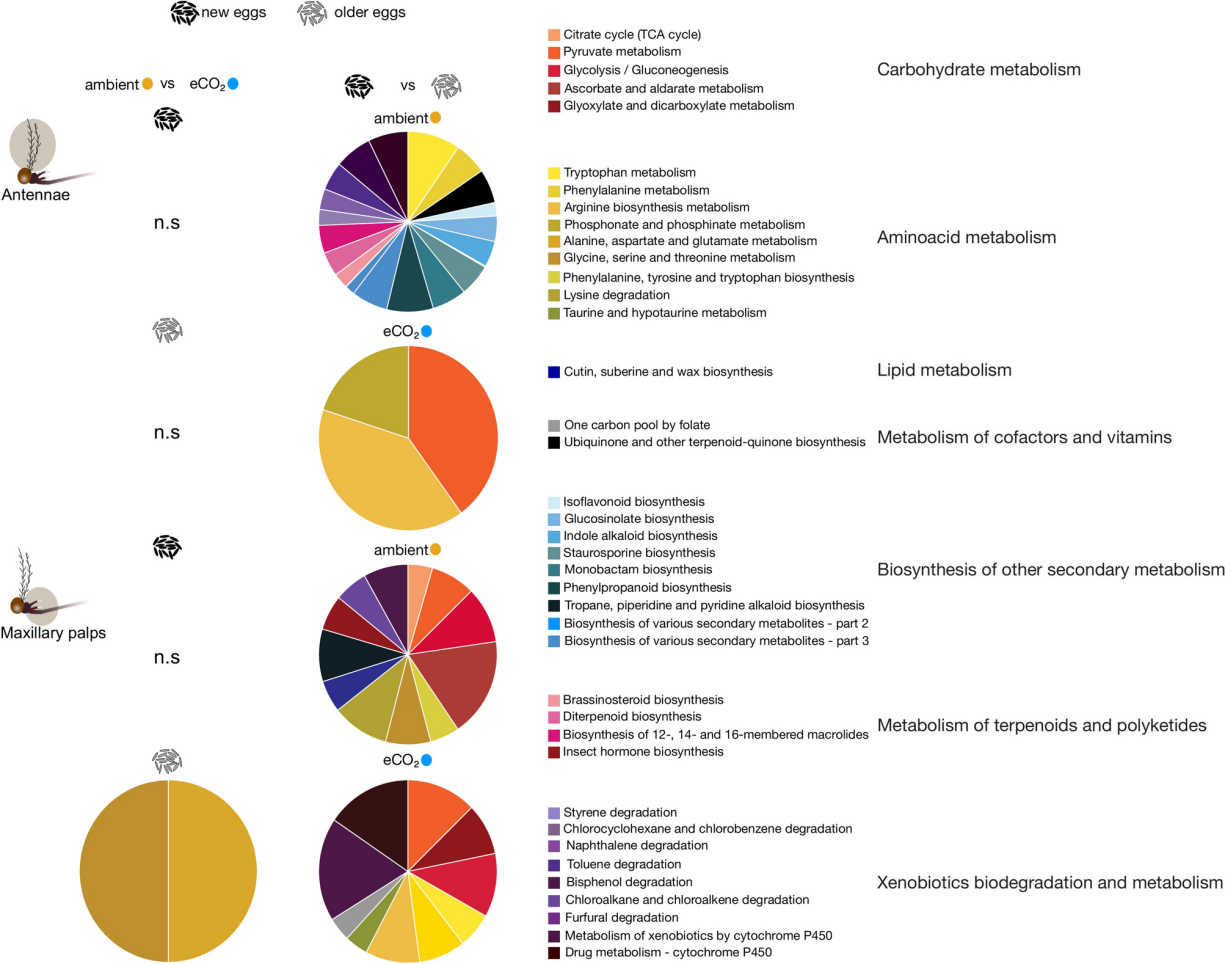
Kyoto Encyclopedia of Genes and Genomes pathway analysis of differentially expressed genes in the antennae and maxillary palps of *Aedes aegypti*. The olfactory tissues were collected from females reared under ambient and elevated CO_2_ conditions, as well as short and extended egg quiescence duration, referred to as new and older eggs, respectively. Pairwise comparisons are arranged in a matrix in relation to the response to CO_2_ conditions (eCO_2_) and egg quiescence period. The categories are annotated from Vectorbase and further classified into pathways designated by Kyoto Encyclopedia of Genes and Genomes database (https://www.genome.jp/kegg/).

The KEGG pathway analysis identified 39 unique metabolic pathway terms, 17 of which were from the metabolism pathways, 9 from biosynthesis of secondary metabolites, four from metabolism of terpenoids and polyketides, and nine identified in the xenobiotic biodegradation pathway (Fig. 3). In the antennal libraries, four DEGs were categorised as xenobiotic response pathway in females reared under ambient CO_2_ conditions, in response to extended egg quiescence. Moreover, in the maxillary palp, six and 28 DEGs contributed to the xenobiotic biodegradation pathway, when reared under ambient and elevated CO_2,_ in response to extended egg quiescence (Fig. 3, Supplementary Table S3). Within the xenobiotic response pathway, several stress response genes, including cytochrome P450 and UDP-glycosyl transferases, were represented across the comparisons in relation to CO_2_ conditions and egg quiescence period.

### Regulation of peripheral chemosensory genes

Elevation in CO_2_ and extended egg quiescence period differentially modulated the expression profile of chemosensory genes, with the highest differential regulation occurring in the maxillary palps.

#### Odorant receptors

**Fig. 4:**
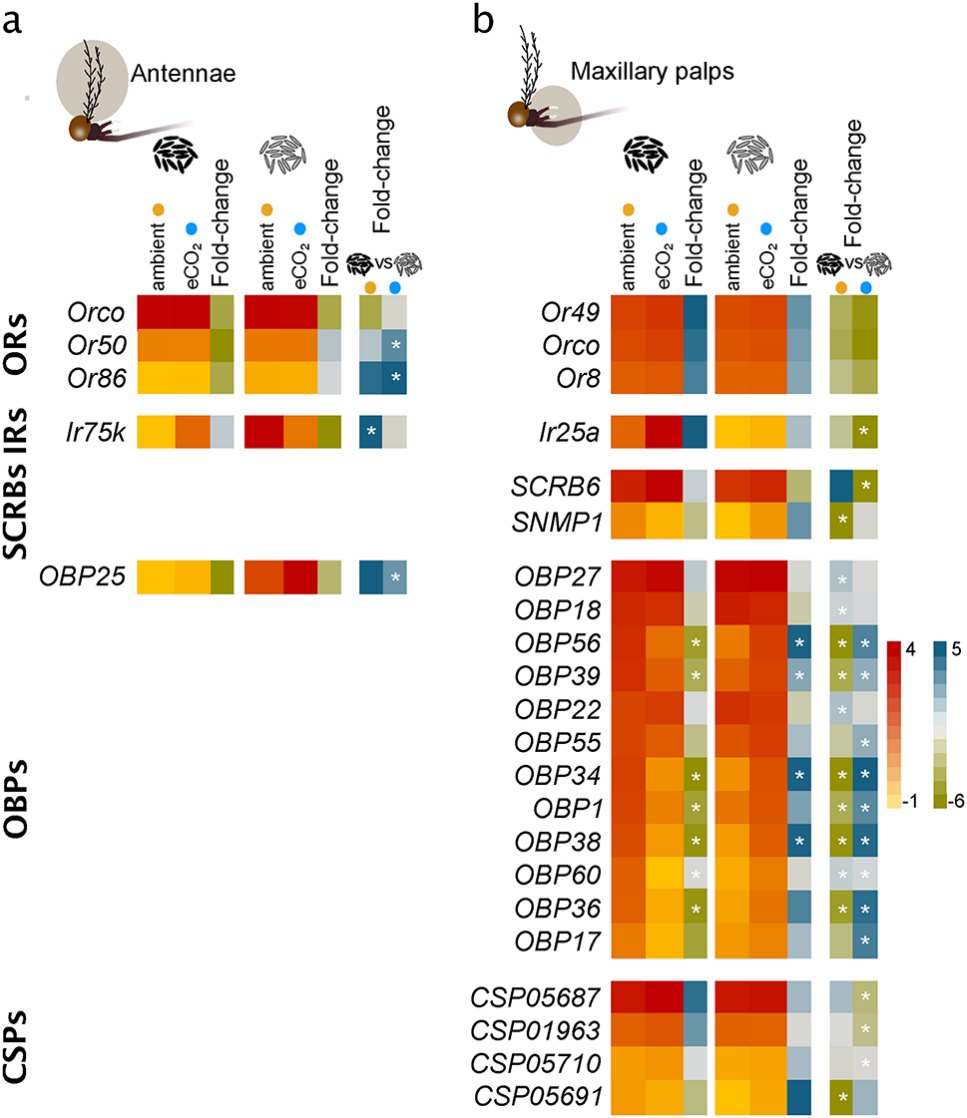
Differential abundance of chemosensory genes in *Aedes aegypti* in response to elevated CO_2_ conditions and extended egg quiescence period. The olfactory tissues were collected from females reared under ambient and elevated CO_2_ conditions, as well as short and extended egg quiescence duration, referred to as new and older eggs, respectively. The abundance of reliably expressed (> 1 transcript per million) chemosensory genes compared between ambient and elevated CO_2_ (eCO_2_) levels, as well as egg quiescent periods, in the antennal (**a**) and maxillary palp (**b**) libraries, and demonstrated by fold-change (> 1.5-fold change; FDR > 0.05). Asterisks on fold change denote significant differences between pairwise comparisons.

Among the 97 annotated *Or*s, 88 and 3, including *Orco*, were reliably expressed in the antennae and maxillary palps of female *Ae. aegypti,* respectively (Supplementary Table S4). While *Orco* was not significantly regulated, the antennally-expressed *Or50* and *Or86* significantly increased in abundance in females emerging from older eggs, in response to elevated CO_2_ conditions (Fig. 4a). The three *Or*s expressed in the maxillary palps were not regulated in response to an elevation in CO_2_ or egg quiescence period (Fig. 4b).

#### Ionotropic receptors

Of the 52 annotated *Ir*s, 33 and 4 were reliably expressed in the antennal and maxillary palp libraries, respectively (Supplementary Table S4). The three co-receptors were reliably expressed, with *Ir25a* having a significantly lower abundance in maxillary palps of females originating from eggs that underwent an extended egg quiescence period and then reared under elevated CO_2_ conditions (Supplementary Table S4, Fig. 4b). Of the 30 tuning *Ir*s expressed in the antennal libraries, *Ir75k* increased in abundance in response to an extended egg quiescence period, when females were reared under ambient CO_2_ conditions (Fig. 4b).

#### Gustatory receptors

Among the 41 annotated *Gr*s, 9 and 5 were reliably expressed in the antenna and maxillary palps libraries, respectively (Supplementary Table S4). No *Gr*s were differentially regulated in the antennal or maxillary palp libraries (Supplementary Table S4).

#### Non-canonical chemoreceptor-related families

Of the 14 annotated *Trp*s, 7 and 6 were reliably expressed in the antennal and maxillary palp libraries, respectively, none of which were differentially regulated (Supplementary Table S4). Similarly, of the 46 annotated *pickpocket* genes, 15 and 9 were reliably expressed in the antennal and maxillary palp libraries, respectively, none of which showed differential expression in response to elevated CO_2_ conditions or egg quiescence period (Supplementary Table S4).

The genes coding for sensory neuron membrane proteins (SNMPs), of which *SNMP1* and *SNMP2* were among the 10 and 11, out of the 13 annotated, reliably expressed *SCRB*s in the antennal and maxillary palp libraries, respectively (Supplementary Table S4). The expression of *SCRB6* and *SNMP1* was downregulated in the maxillary palps of females reared under elevated and ambient CO_2_ conditions, respectively, in response to extended egg quiescence period (Fig. 4b).

#### Soluble odorant-binding proteins

The genes encoding for OBPs and CSPs were highly abundant in the antennae and maxillary palps libraries. Out of the 52 annotated *OBP*s, 33 and 35 were reliably expressed in the antennal and maxillary palp libraries, respectively (Supplementary Table S4). Only one *OBP*, *OBP25*, increased in abundance in the antennae of females reared under elevated CO_2_ conditions in response to an extended egg quiescence period (Fig. 4a). In the maxillary palp libraries, *OBP*s were differentially regulated in response to elevated CO_2_: seven *OBP*s were significantly lower in abundance in females emerging from new eggs, while four *OBP*s were higher in abundance in females emerging from older eggs, in response to elevated CO_2_ conditions (Fig. 4b). In response to extended egg quiescence period, the abundance of *OBP*s were differentially regulated in relation to CO_2_ condition: seven out of the ten differentially expressed *OBPs* in the maxillary palp libraries of females reared under ambient CO_2_ conditions were lower in abundance, while nine *OBP*s were higher in abundance in females reared under elevated CO_2_ conditions (Fig. 4b).

Out of the 17 annotated *CSP*s, seven and ten were reliably expressed in the antennal and maxillary palp libraries, respectively (Supplementary Table S4). The *CSP*s did not display any differential expression in response to CO_2_ conditions and egg quiescence period in the antennal libraries. However, in the maxillary palp libraries, one and three *CSP*s decreased in abundance in females when reared under ambient and elevated CO_2_ conditions, respectively, in response to an extended egg quiescence period (Fig. 4b).

## Discussion

Based on this transcriptome analysis, the effect of an elevation in CO_2_ level, to that predicted under extreme conditions^7^, appears to be gene-family specific, while egg quiescent duration has a distinct and overall impact on gene expression, particularly in the maxillary palp. Differential expression of genes in both antennae and maxillary palps involved in metabolism and xenobiotics emphasise a stress response as a consequence of elevated CO_2_ and extended egg quiescence duration, similar to the systemic response shown in other insects to environmental stressors^56^. Contrasting regulation of select members of chemosensory gene families, ORs, IRs, SNMPs, OBPs and CSPs, in the antennae and maxillary palp, may regulate the observed differences in resource-seeking behaviour in response to the two external stressors in female *Ae. aegypti*^6^. Overall, this study provides insights into how environmental stress impacts the peripheral olfactory system of insects and ensuing behaviour.

The differential feeding behaviour of *Ae. aegypti* as a result of different egg quiescence durations, and when reared under elevated CO_2_^6^, while appearing to have no significant generalised effect on gene expression, is likely a result of more targeted regulation of genes as indicated in the GO slim and KEGG analyses. The high number of significant DEGs, characterised by GO slim analysis, emphasises an interactive effect of elevated CO_2_ conditions and extended egg quiescence period on gene regulation in the peripheral olfactory system. The differentially regulated genes, predominantly in the maxillary palp, divides into categories including energy metabolism and xenobiotic response pathways, which is highlighted through KEGG analysis, and emphasises a significant transcriptional regulation of stress-induced genes in an organ that is involved in the detection of CO_2_ and other host-related chemosensory signals^57,58^. A similar transcriptional regulation of metabolic genes, in response to elevated CO_2_, has been demonstrated in aquatic invertebrates and insects^59,60^. Tissue-specific effect on gene expression regulation in the olfactory system, in response to elevated CO_2_, has also been demonstrated in salmon^61^. While elevated CO_2_ levels do not appear to directly trigger the xenobiotic response pathways, elevated CO_2_ upregulates the transcription of genes encoding for detoxifying enzymes, including cytochrome P450s^62,63^, [this study]. Xenobiotic response genes, including members of the cytochrome P450 family, are regulated in response to a variety of environmental stressors, including volatile compounds^64,65^, prolonged exposure to insecticides^66,67^, and abiotic stressors^68–70^. Cytochrome P450s act as odorant degrading enzymes in the insect peripheral olfactory system^71^. Hence, the oxidative stress and potential acidification of the sensillum lymph, as a result of the conversion of CO_2_ into carbonic acid^72^, may explain the observed response in this degradation pathway. Acidification of the sensillum lymph influences the folding of OBPs^73–75^, which can lead to alterations in protein function. Although mosquitoes acid–base regulate under varying pH conditions^76,77^, it remains unclear how the buffering capacity is impacted by prolonged exposure to elevated CO_2_. Furthermore, how this affects the membrane-bound receptors^78^, and the cascading effects on neuronal signalling^79,80^, remains to be studied.

Elevated CO_2_ levels, accentuated by egg quiescence duration, differentially affected the expression of soluble and membrane-bound chemosensory genes, which may directly affect the behaviour of disease-transmitting mosquitoes^6^, [this study]. Of the soluble odorant-binding proteins, insect OBPs facilitate odorant transport, odorant-receptor interactions and gain control^81^, as well as xenobiotic adaptations^82^. The significant differential regulation of *OBP*s, predominantly in the maxillary palp, emphasises the important role of these genes in response to elevated CO_2_ levels and extended egg quiescence duration. Of the 12 differentially regulated *OBP*s, only *OBP22* and *OBP39* have been functionally characterised, and demonstrated to detect long-chain fatty acids involved in host- and oviposition-site seeking, respectively^83,84^. The abundance of a subset of *OBP*s, including *OBP56, OBP39, OBP34* and *OBP38,* shifted in response to elevated CO_2_, from low in new eggs to high in older eggs, suggesting a conserved regulatory pathway for these OBPs in response to stress. Considering the role of OBPs, the demonstrated regulation of genes will likely affect the interaction between odorant ligands and the membrane-bound receptors.

Among the membrane-bound receptors, the differential regulation of *Or*s in the antenna provides an insight into the regulatory mechanism regulating *Or* expression in response to environmental stress^85^, despite the unknown functional relevance of these changes for *Ae. aegypti*^44,86–89^. The absence of regulation in other *Or*s in both antennae^53,90^ and maxillary palps^91^ suggests that core Or-mediated sensory detection remains largely unaffected, as is the case for other membrane-bound receptors. Among the differentially regulated *Ir*s that have been functionally characterised, the Ir co-receptor *Ir25a* is involved in the detection of amines^92,93^, whereas the tuning Ir, *Ir75k*, is sensitive to short-chain carboxylic acids^94^. These chemical classes play important roles in host- and oviposition-site selection^95,96^, and the differential regulation of the receptors detecting these odorants may affect the efficient resource seeking by mosquitoes. In *Drosophila*, *Ir25a* is required for context-dependent attraction to CO_2_^97^, and in female *Ae. aegypti, Ir25a* is co-expressed in the maxillary palp CO_2_ sensitive OSN^38^. The functional significance of the lower abundance of *Ir25a* in response to stress requires further investigation. While short-term exposure to elevated CO_2_ significantly impact host seeking, as a consequence of sensory constraint^21^, the genes encoding for the subunits forming the CO_2_ receptor^98^ were not regulated in response to developmental exposure to high CO_2_. Whether long-term exposure to high CO_2_ levels has a similar affect, and how this is regulated is yet unknown. Taken together, the interaction of elevated CO_2_ and extended egg quiescence differentially affect the expression of chemosensory genes that likely play key roles in regulating mosquito behaviours.

This study provides evidence that anthropogenic climate change factors, such as elevated CO_2_, interact with other stress factors, such as egg quiescence duration, elicit a stress response in the peripheral olfactory system of mosquitoes and that the capacity of females to detect ecologically-relevant volatile organic compounds may be hampered. While previous studies have demonstrated negative effects of elevated CO_2_ and egg quiescence duration on life-history parameters of both aquatic and terrestrial stages of *Ae. aegypti,* and subsequent carry-over effect on the feeding response of adult females, future experiments are required to assess how these stress factors affect odour-mediated behaviour and physiology.

## Methods

### Mosquito rearing and tissue collection

For general colony maintenance, *Ae. aegypti* (Rockefeller) were maintained under 27 ± 2 °C, 65 ± 5% relative humidity and a 12 h: 12 h light: dark cycle. The adults had ad libitum access to 10% sucrose (w/v). Females were blood fed with defibrinated sheep blood (Håtunalab AB, Bro, Sweden), using a membrane feeding system (Hemotek Ltd, Blackburn, UK) for egg production. The eggs, deposited on moist filter paper, were collected, dried, labelled and stored for subsequent experiments to account for different egg quiescent periods. The CO_2_ acclimatization experiments were conducted in two high-precision climate chambers, in which temperature, humidity and light conditions were maintained as above. The CO_2_ concentration in the chambers was 400 ppm (current ambient), and 1000 ppm (elevated CO_2_), respectively, in which pure CO_2_ (Strandmöllen, Ljungby, Sweden) was delivered and mixed into the ventilation system. Age-controlled eggs (2-week or 3–6-month quiescent periods) were introduced to each experimental chamber, in which eggs from the same cohort were divided equally between two chambers, resulting in a larval density of 100 larvae per 600 ml of water, in each rearing tray. The larvae were fed with fish food **(**TetraMin® Flakes, Melle, Germany) daily (1 mg larvae^-1^), normalised for larval mortality. Upon pupation, individual pupae were collected into small (30 ml) plastic cups with distilled water and placed into Bugdorm cages (17.5 cm × 17.5 cm × 17.5 cm; Megaview Science Co., Ltd, Taichung, Taiwan). The emerging adults had ad libitum* acce*ss to water until tissue was collected.

Collection of teneral (30 ± 6 h) female antennae and maxillary palp were done at Zeitgeber time 10–12, *i.e.*, the peak diel activity period of *Ae. aegypti*^99^. For the dissection, females were anesthetised on ice, and the tissues removed using a pair of fine sterilised forceps, with separate pairs of forceps used for each olfactory tissue type, and then placed into 200 µl of RNAlater (Thermo Fisher Scientific, Gothenburg, Sweden). Forceps were sterilised in between each biological replicate using 70% ethanol. The tissue was stored at room temperature overnight, then at -20 °C overnight, and thereafter at -80 °C until RNA extraction. A total of 16 antennal libraries were generated, with each library comprising pooled tissues from 50 individuals per replicate from different cohorts, across two CO₂ conditions, two egg quiescence periods, and four biological replicates (50 tissues × 2 CO₂ levels × 2 quiescent periods × 4 replicates = 800 pairs of tissues). Similarly, 16 maxillary palp libraries were constructed using the same pooling strategy, yielding an additional 800 pairs of tissues. In total, 1,600 pairs of tissues were collected for all (32) libraries.

### RNA extraction and sequencing

Total RNA extraction was performed using the RNeasy microRNA kit (Qiagen, Hilden, Germany) following the manufacturer’s protocol with an additional step of quick freezing with liquid nitrogen to facilitate the homogenisation of the tissues. The RNA extracted was immediately stored at -80 °C and later quantified using the TapeStation system 4150 (Agilent Technologies, Inc, Santa Clara, US). The samples were shipped on dry ice to Eurofins Genomics (Constance, Germany), where INVIEW ultra-low transcriptome libraries were constructed using NovaSeq Illumina genome sequencing technology (Illumina NovaSeq 6000 S4 PE150 XP). The cDNA library construction was realised using Eurofins proprietary protocol, generating 2 × 150 bp coverage paired-end reads with a depth of 20 million paired-end reads (Supplementary Table S5).

### Read mapping and annotation

Prior to the quantitative assessment of the library sequences, the samples underwent quality control steps involving the removal of adapter sequences, and discarding sequences with a Phred score of below 40, using CLC Genomics Workbench (23.0.5, Qiagen, Aarhus, Denmark). Three libraries were removed from further analysis due to cross-contamination between tissues (Supplementary Table 1)^100^. The sequences were mapped to the *Ae. aegypti* reference genome (VectorBase: *Aedes aegypti* LVP_AGWG, AaegL5.3).

### PCA analysis

Principal component analysis (PCA) was performed to estimate the effect of elevated CO_2_ and egg quiescence period on the overall expression profile. The high-dimensional dataset containing the antennal and maxillary palp libraries was projected onto two-dimensional components to determine the variance between libraries using the toolbox for RNA-seq data in CLC Genomics Workbench. The individual and interactive effect of CO_2_ level and egg quiescent period on each olfactory tissue was assessed through permutational multivariate analysis of variance (PERMOVA) using “adonis2” function under the *vegan* package in RStudio.

### RNA seq and differential expression analysis

For the transcriptome analysis, transcripts per million (TPM) was used, with a reliable expression of genes determined to be above a threshold of 1 TPM. Differential transcript abundance was analysed using a negative binomial distribution with a gamma-Poisson mixed distribution in CLC Genomics Workbench (https://digitalinsights.qiagen.com/). To account for false positives during the statistical tests, the false discovery rate (FDR) with p-value correction was performed using the Benjamin-Hochberg method^101^. The analysis generated a mean abundance value, fold change (FC) and FDR p-values that were accessed for differential expression. Genes were considered significantly differentially expressed when fold change > 1.5 and FDR p-value < 0.05.

### Functional enrichment analyses

To assess the effects of elevated CO_2_ and extended egg quiescence period on molecular function level and metabolic pathways, a gene ontology (GO) analysis and KEGG (Kyoto Encyclopedia of Genes and Genomes) analysis were performed. The GO and KEGG terms used for the identified differentially expressed genes (DEGs) in the antennae and maxillary palps, were identified from VectorBase (AaegL5.3, Release 68). The VectorBase GO enrichment tool was used for assessing the molecular function, with both computed and curated evidence limited to GO slim terms. The VectorBase metabolic pathway enrichment was used for KEGG analysis. The significance cut-off was set to alpha (α) = 0.05.

## Supplementary Information

Supplementary Information 1.

Supplementary Information 2.

Supplementary Information 3.

Supplementary Information 4.

Supplementary Information 5.

Supplementary Information 6.

## Data Availability

All data generated are presented in the publication. The transcriptome data generated and analysed during this study is available in the NCBI project database, with BioProject ID: PRJNA1195965. https://www.ncbi.nlm.nih.gov/sra/PRJNA1195965.
