# Supplementary material for: Impact of elevated CO2 level and egg quiescence duration on gene expression in the peripheral olfactory system of Aedes aegypti
Source: Sci Rep. 2025 Apr 24;15:14318. doi: 10.1038/s41598-025-98159-w (PMC12022256; doi:10.1038/s41598-025-98159-w)
Supplement: Supplementary file 1 — Supplementary Information 1. [file 41598_2025_98159_MOESM1_ESM.pdf]

Supplementary Files

Supplementary Table S1. The abundance of genes in the antennal and maxillary palp libraries (in TPM) for ambient and elevated CO_2 conditions, as well as the two egg quiescence periods. Fold change and false discovery rate (FDR) p-value are represented.

Supplementary Table S2. The abundance of core eukaryotic gene transcripts (in TPM) across antennal and maxillary palp libraries in response to elevated CO_2 conditions and extended egg quiescence duration.

Supplementary Table S3. Raw data from the Kyoto Encyclopedia of Genes and Genomes analysis conducted in Vectorbase, represented for all significant comparisons concerning CO_2 conditions and egg quiescence duration.

Supplementary Table S4. Abundance of chemosensory genes (in TPM), including Ors, Irs, Grs, OBPs, CSPs, SCRBs and non-canonical chemosensory proteins. Fold change and false discovery rate (FDR) p-value are represented.

Supplementary Table S5. RNA-seq reads of antennal and maxillary palp libraries.

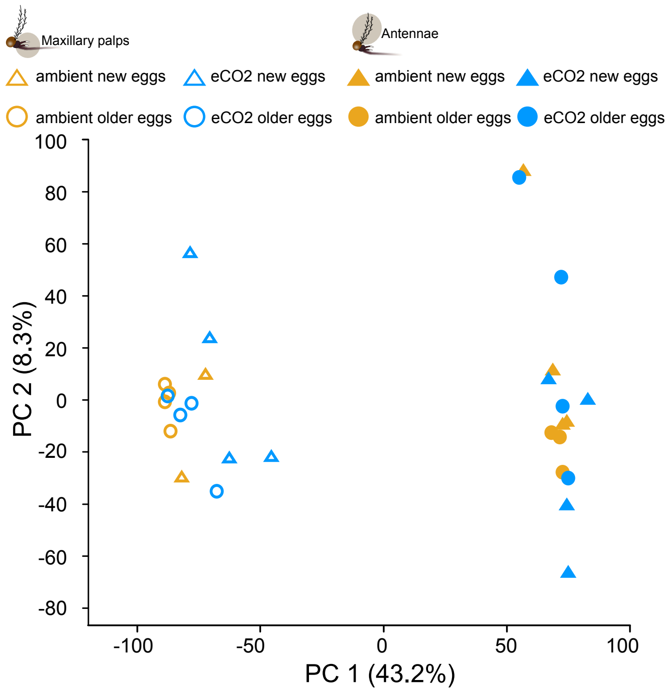

Supplementary Figure S1. Principal component analysis of antennal and maxillary palp libraries of teneral females emerging from new and older eggs, with short and extended egg quiescent duration, respectively, reared under ambient and elevated CO_2 conditions. A total of 29 libraries were analysed to estimate the change in overall gene expression.
